# Supplementary material for: The interactome of intact mitochondria by cross-linking mass spectrometry provides evidence for coexisting respiratory supercomplexes
Source: Mol Cell Proteomics. 2017 Dec 8;17(2):216–32. doi: 10.1074/mcp.RA117.000470 (PMC5795388; doi:10.1074/mcp.RA117.000470)
Supplement: Supplemental Data [file supp_RA117.000470_133922_0_supp_23978_fzffwf.zip › spectra_annotation/mito_DR_spectra_annotation/342-1-2-1-3-1.pdf]

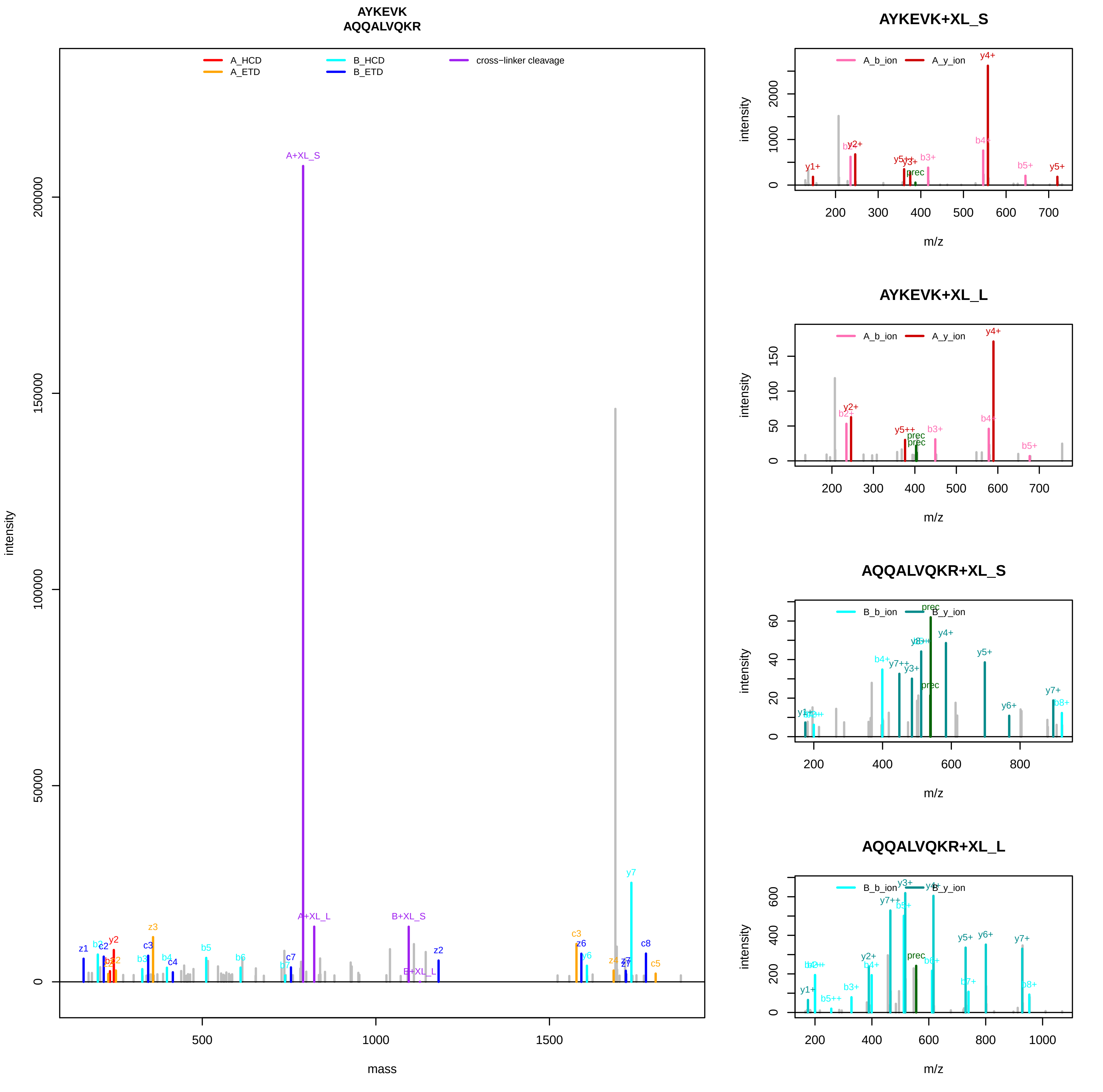
AYKEVK
AQQALVQKR
A_HCD
A_ETD
B_HCD
B_ETD
cross−linker cleavage
A+XL_S
A+XL_L
B+XL_S
B+XL_L
intensity
mass
0
50000
100000
150000
200000
500
1000
1500
z1
b2
c2
y2
c3
b3
z3
b4
c4
b5
b6
c7
b7
c3
z6
y6
z4
z7
y7
c8
c5
z2
AYKEVK+XL_S
A_b_ion
A_y_ion
y4+
b4+
b2+
y2+
y5++
y3+
b3+
prec
y1+
b5+
y5+
m/z
AYKEVK+XL_L
AQQALVQKR+XL_S
B_b_ion
B_y_ion
y8++
y4+
b4+
y7++
y3+
y5+
y6+
y7+
b8+
y1+
AQQALVQKR+XL_L
y3+
y4+
y7++
b5+
y2+
b3+
b5++
b6+
b7+
y5+
y6+
b8+
